# Discovery of a Novel Coronavirus in Swedish Bank Voles (*Myodes glareolus*)

**DOI:** 10.3390/v14061205

**Published:** 2022-06-01

**Authors:** Anishia Wasberg, Jayna Raghwani, Jinlin Li, John H.-O. Pettersson, Johanna F. Lindahl, Åke Lundkvist, Jiaxin Ling

**Affiliations:** 1 Zoonosis Science Center, Department of Medical Biochemistry and Microbiology, Uppsala University, 751 23 Uppsala, Sweden; anishia.wasberg@imbim.uu.se (A.W.); john.pettersson@imbim.uu.se (J.H.-O.P.); johanna.lindahl@imbim.uu.se (J.F.L.); ake.lundkvist@imbim.uu.se (Å.L.); 2 Department of Zoology, University of Oxford, Oxford OX1 4BH, UK; jayna.raghwani@zoo.ox.ac.uk; 3 Department of Medical Biochemistry and Microbiology, Uppsala University, 751 23 Uppsala, Sweden; jinlinli@whu.edu.cn; 4 Sydney Institute for Infectious Diseases, School of Life and Environmental Sciences and School of Medical Sciences, The University of Sydney, Sydney, NSW 2006, Australia; 5 Department of Biosciences, International Livestock Research Institute, Nairobi 00100, Kenya; 6 Department of Clinical Sciences, Swedish University of Agricultural Research, 750 07 Uppsala, Sweden

**Keywords:** coronavirus, bank voles, RNA-sequencing, prevalence

## Abstract

The unprecedented pandemic COVID-19, caused by severe acute respiratory syndrome coronavirus 2 (SARS-CoV-2), with bats as original reservoirs, has once again highlighted the importance of exploring the interface of wildlife diseases and human health. In this study, we identified a novel *Betacoronavirus* from bank voles (*Myodes glareolus*) in Grimsö, Sweden, and this virus is designated as Grimso virus. Repeated detection over three years and an overall prevalence of 3.4% suggest that the virus commonly occurs in bank voles. Furthermore, phylogenetic analyses indicate that the Grimso virus belongs to a highly divergent *Embecovirus* lineage predominantly associated with bank voles. Given that bank voles are one of the most common rodent species in Sweden and Europe, our findings indicate that Grimso virus might be circulating widely in bank voles and further point out the importance of sentinel surveillance of coronaviruses in wild small mammalian animals, especially in wild rodents.

## 1. Introduction

Coronaviruses (CoVs) have a high plasticity in host infection which produces a great diversity of CoVs and a complexity evolution. Zoonotic resources of human pathogenic CoVs are mostly from bats or rodents. Coronavirus (CoV) belongs to the Coronaviridae family in the Nidovirales order. They are the largest known RNA viruses with a genome size of 26–32 kb. CoVs have the ability of zoonotic transmission to humans or livestock, and they can cause respiratory or enteric diseases. There are four genera within the coronavirus family, the alpha-, beta-, gamma- and delta-coronaviruses. The genera alpha- and beta-coronaviruses are usually associated with human health of importance [1].

Before the emergence of SARS-CoV, MERS-CoV and SARS-CoV2, four human CoVs HCoV-OC43, HCoV-HKU1, HCoV-NL63, and HCoV-229E, have been adapted to the human population, which were responsible for approximately 30% of endemic common colds in human [1]. Two of these human pathogenic CoVs, HCoV-OC43 and HCoV-HKU1, have the most common ancestry with rodent-borne CoVs based on phylogenetic analyses, indicating that rodents are most likely the reservoirs, and more importantly, the plausibility for rodent CoVs spilling over and infecting humans [2].

Bank voles (*M. glareolus*) is one of the most common rodent species in Europe and a known reservoir for several zoonotic pathogens, such as *Puumala orthohantavirus* and *Francisella tularensis*. Previous studies have detected various *Alphacoronavirus* and *Betacoronavirus* in bank voles in the UK, Poland, Germany, and France [3,4]. Here, following a virome investigation of Swedish bank voles collected in Grimsö, Sweden, we report two complete genome sequences of the Grimso virus, along with its evolutionary relationship to other rodent CoVs and its prevalence over a three-year study period.

## 2. Materials and Methods

### 2.1. Rodent Samples Collection

A total of 450 bank voles were sampled at the same site in Grimsö, Sweden (59°43′ N, 15°28′ E) between 2015 and 2017. All trapping and sampling were approved by the Animal Experiment Ethical Committee, Umeå (Reference: A13–14), and followed the Swedish Board of Agriculture regulations. Animal species were identified in the laboratory, and lung tissues were harvested and subsequently stored at −80 °C until further investigation, including molecular species identification as described in [5].

### 2.2. RNA Extraction and Reverse Transcription-PCR (RT-PCR)

Total RNA was extracted using Qiagen RNeasy mini kit (Qiagen, Hilden, Germany). Specific primers targeting the spike protein gene (CoVF: 5′-Ggtcaaactactgaatttattg-3′, CoVR: 5′-Aatccatcagaaccaacgac-3′) were designed based on a virome investigation of Swedish bank voles previously. This primer set was used for screening coronaviruses in 266 bank voles captured from Grimsö between 2015 and 2017. In parallel, we also screened the same samples using a published pan-coronavirus RT-PCR [6]. Positive samples were sent for Sanger sequencing at Macrogen Europe (https://dna.macrogen-europe.com/eng/ (accessed on 1 June 2021)).

### 2.3. Next-Generation Sequencing and Sequence Assembly

Two positive RNA samples extracted from lung tissues (Grimso215 and Grimso2306) were sent for RNA sequencing, at the Illumina NovaSeq 6000 sequencing platform, from Novogene Hong Kong (https://en.novogene.com/ (accessed on 1 May 2021)). The number of raw reads reached around 50 million pair-end reads of 150 base-pairs (bp). A data analysis pipeline was used to trim and assemble the reads from the sequencing results as described in [7]. We obtained 86,322,748 (96.89% of raw reads) and 105,068,356 (98.81% of raw reads) clean pair-end reads of 150 base-pairs (bp) after filtering from Grimso215 and Grimso2306 samples, respectively. Full-genome and subgenomic sequences of the strain Grimso215 were obtained through de novo assembly using Trinity v2.13.2 with default settings [8]. Using the de novo assembled full genome sequence as a reference, we mapped the reads from the two samples separately (strain Grimso215: 104,891 reads; strain Grimso2306: 2700 reads). As a result, we obtained complete and near-complete coronavirus genome sequences from the strain Grimso215 (100%; 31,317 nt, with a mean coverage value of 502) and strain Grimso2306 (98.2%; 30,767 nt, with a mean coverage of 12.9), respectively. The genome sequences are available via NCBI GenBank (accession number: OM373090 and OM373091).

### 2.4. Phylogenetic Analysis

**Figure 1:**
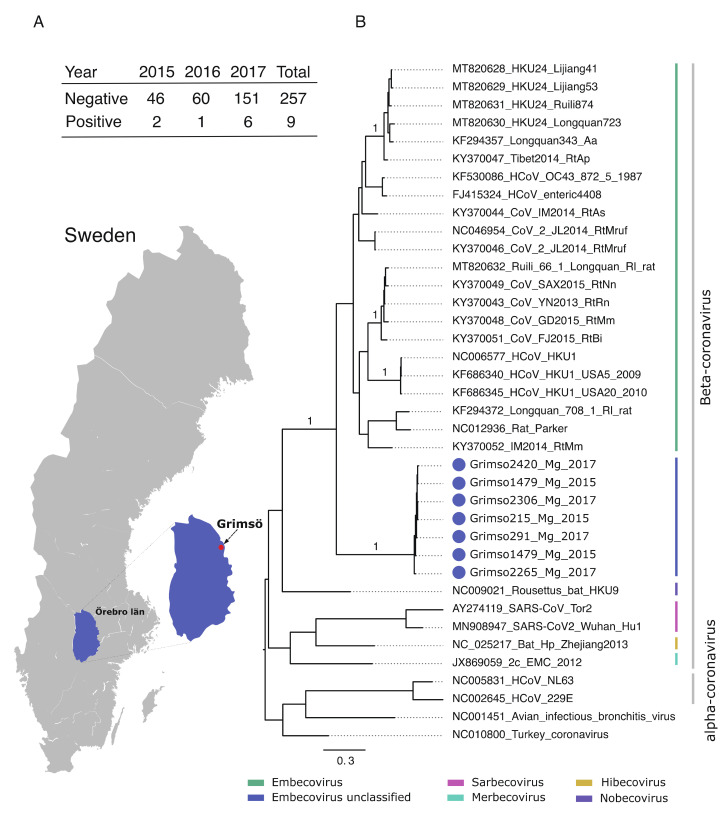
(**A**) Geographic map showing the province Örebro, Sweden and the site of sampling (Grimsö) where bank voles were captured. Table demonstrating the prevalence of Grimso virus from 2015 to 2017. (**B**) MrBayes midpoint root tree based on the 252 nt of the spike gene. The scale bar indicates the nt substitution per site. The numbers above the branches indicate the posterior probability. Grimso virus samples are highlighted in blue.

**Figure 2:**
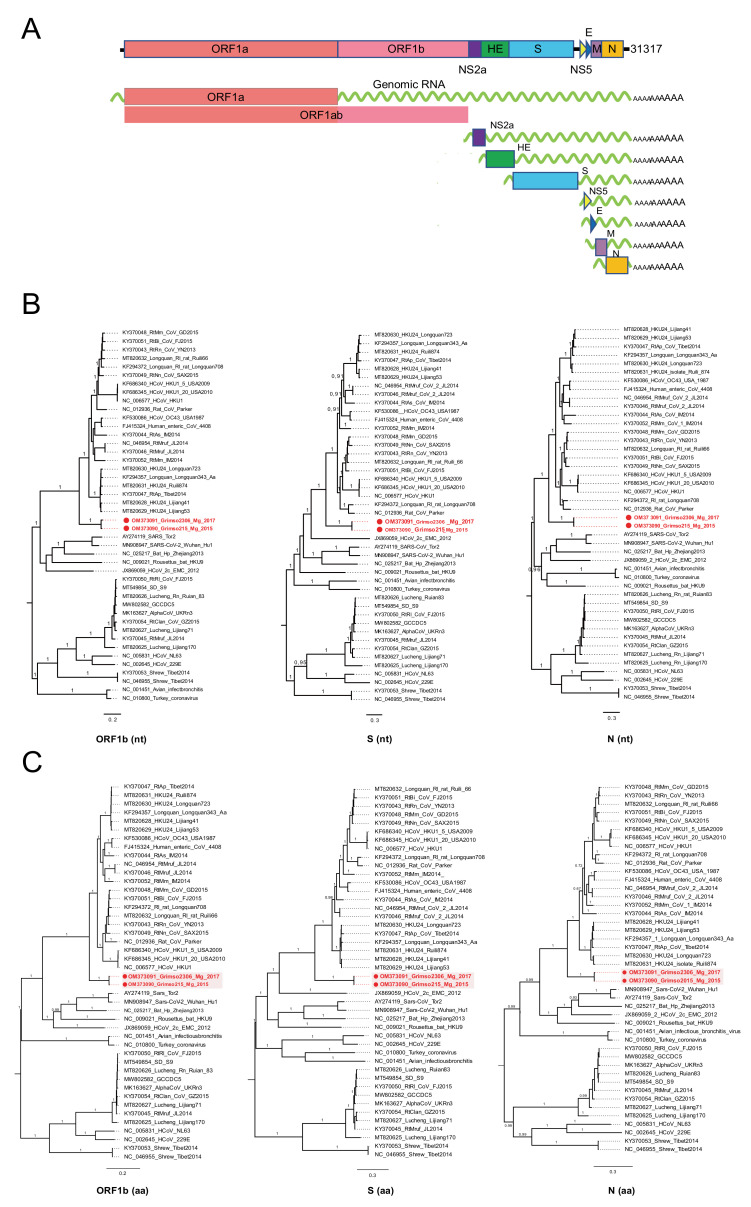
(**A**) Genomic RNA and subgenomic mRNAs organizations of Grimso virus, Grimso215 strain. MrBayes trees based on the complete nucleotide sequences (**B**) and amino acid sequences (**C**) of ORF1b, S, and N genes of CoVs. The red color shows Grimso virus. The scale bar indicates the nt and aa substitution per site, respectively. The numbers above the branches indicate the posterior probability.

**Figure 3:**
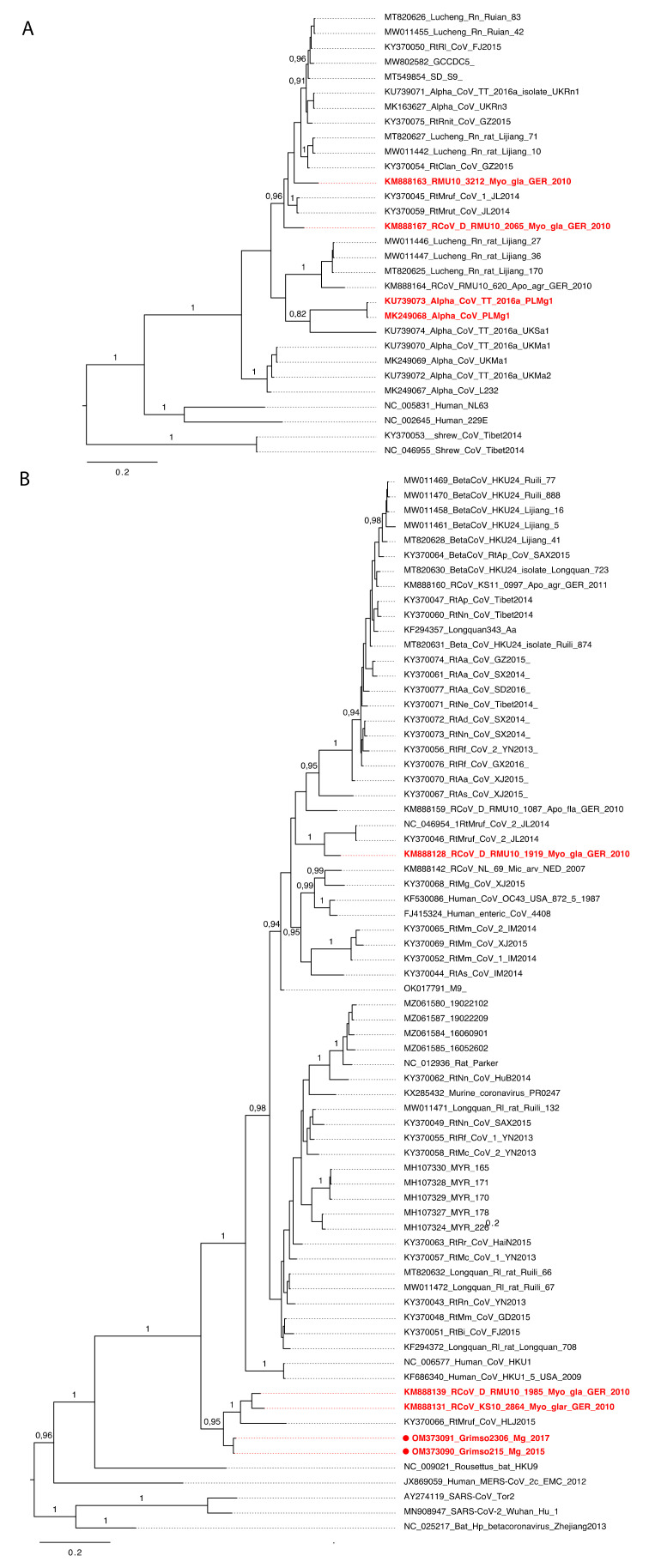
MrBayes tree based on 441nt of partial RdRp gene of CoVs. (**A**) Phylogeny of alphacoronaviruses and (**B**) betacoronaviruses. The red color shows CoVs carried by bank voles across Europe. The scale bar indicates the nt substitution per site. The numbers above the branches indicate the posterior probability.

For the recombination and phylogenetic analyses, the reference rodent CoVs were downloaded from the NCBI RefSeq database, and multiple sequence alignment was obtained using MAFFT v7.490, which was refined using trimAl v.1.4.1 [9,10]. Pairwise genetic distances were obtained using Geneious Prime v.2019.2.1. Potential recombination events were detected by using Simplot v. 3.5.1 and RDP3 [11]. We assembled six multiple nucleotide sequences alignments for the tree inference, including (A) thirty-one partial spike protein gene sequences from NCBI RefSeq viral database and seven sequences from this study (Figure 1), (B) ORF1b gene, (C) spike protein gene, and (D) nucleocapsid protein gene from forty-two coronavirus nucleotide and amino acid sequences from the database and two genome sequences from this study (Figure 2B,C); (E) thirty partial RdRp gene sequences from alphacoronavirus genome sequences retrieved from the database (Figure 3A); (F) sixty-nine partial RdRp gene sequences from betacoronavirus genome sequences retrieved from the database and two sequences from this study (Figure 3B). The substitution model was determined by using jModelTest2 [12]. Phylogenetic trees were built using MrBayes rooted on the midpoint [13]. All computational calculations from this study were performed using the UPPMAX service from Uppsala University (https://www.uppmax.uu.se/ (accessed on 20 April 2022) (Project ID: SNIC 2019/8-68)).

### 2.5. Protein Domain Analysis

To analyze possible receptor usages, the sequences of spike protein and hemagglutinin esterase genes were examined by searching in the blastx with default settings.

## 3. Results

### 3.1. Prevalence of Grimso Virus

Initially, we screened 266 bank voles collected during 2015–2017 at the same site in Grimsö, Sweden (59°43′ N, 15°28′ E) for coronaviruses using an in-house PCR method based on custom primers targeting the spike gene (Figure 1). We detected nine positive samples, which we further characterized using Sanger sequencing. We also screened the same samples using a published pan-coronavirus RT-PCR [6], although this failed to detect any coronaviruses.

Figure 1 shows the location of the study site and demonstrates the prevalence of the Grimso virus between 2015 and 2017. We obtained partial spike gene sequences (252 nt) from seven out of nine positive samples using Sanger sequencing. Pairwise genetic analyses indicated that the partial Grimso virus sequences shared 98.0–100% and 94.0–100% sequence identity at the nucleotide and amino acid levels, respectively. Strikingly, Grimso virus sequences showed less than 60% and less than 50% sequence identity at both the nucleotide and amino acid levels, respectively, with other rodent Betacoronaviruses. Bayesian phylogenetic analysis of partial spike gene sequences from thirty-one reference CoV genome sequences indicates that the Grimso virus sequences form a distinct monophyletic group that cluster with other rodent-borne Embecoviruses with strong statistical support (posterior probability = 1.0).

### 3.2. Genome Organization of Grimso Virus

Thereafter, we selected two samples collected in 2015 (Grimso215) and 2017 (Grimso2306) for RNA-sequencing to characterize the complete genome. We obtained a full-length coronavirus sequence from the bank vole sample Grimso215 (31,317 nt) and a near-complete coronavirus sequence from the bank vole sample Grimso2306 (98%; 30,767 nt). The in-depth phylogeny of Grimso virus is demonstrated in Figure 2 where Figure 2A outlines the whole genome, including seven subgenomic regions, of the Grimso virus, strain Grimso215. The Grimso virus genome encodes for hemagglutinin esterase (HE), spike protein (S), envelop protein (E), membrane protein (M), and nucleocapsid protein (N). We found no significant evidence for recombination in the genome of strain Grimso215. Phylogenetic analyses based on the ORF1b, S and N gene sequences consistently showed that the strains Grimso215 and Grimso2306 fell within known rodent Betacoronavirus diversity and formed a distinct lineage within the subgenus Embecovirus (Figure 2B). Additionally, we analyzed the amino acid sequences of ORF1b, S and N genes, which confirmed the phylogenetic relationship (Figure 2C).

Pairwise sequence identity of Grimso virus (strain Grimso215) with other rodent CoVs ranged from 55 to 79% at the nucleotide level and 44 to 67% at the amino acid level, confirming that the Grimso virus is divergent and genetically distinct from earlier described rodent CoVs (Table 1).

### 3.3. Phylogenetic Analysis

To better understand the evolutionary history of rodent CoVs and the phylogenetic positioning of the divergent Grimso strains, we undertook an additional phylogenetic analysis based on 441 nt of the partial RNA-dependent RNA polymerase (RdRp) gene from 101 CoV genome sequences. The results indicated that bank voles harbor at least three virus species across the *Alphacoronavirus* and *Betacoronavirus* genera (Figure 3A,B). Specifically, four viruses isolated from bank voles in Germany, Poland, and the UK [14] were closely related to the Lucheng Rn rat coronavirus (genus: *Alphacoronavirus*) (Figure 3A). In addition, we also found two distinct Embecoviruses associated with bank voles (Figure 3B): (1) a bank vole coronavirus from Germany closely related to Myodes coronavirus 2JL14 (subgenus: *Embecovirus*) and (2) a cluster of CoVs isolated from bank voles in Germany and France, along with strains Grimso215 and Grimso2306 from this study, which formed separate divergent lineage within the subgenus *Embecovirus* (Figure 3B).

### 3.4. Protein Domain Analysis

We examined the spike protein, hemagglutinin esterase and possible receptor usages by searching in the blastx. It had only specific hits in the S2 region but not in the S1 or receptor-binding regions (Figure S1).

## 4. Discussion

Rodents are the primordial hosts of CoVs, and rodent CoVs constitute at least two subgenera, *Luchacovirus* and *Embecovirus* from genera *Alphacoronavirus* and *Betacoronavirus*, respectively [14,15]. In this study, we have discovered a highly divergent betacoronavirus (Grimso virus) in the Swedish bank voles. Furthermore, based on our knowledge, this study is the first time to identify full-genome features of Grimso virus, together with the prevalence and diversity of this rodent CoV in Sweden.

The Grimso virus is highly divergent and genetically distinct from earlier described rodent CoVs based on the RNA-sequencing results, which also explains that we failed to detect any CoVs by using a published pan-coronavirus RT-PCR. By using the specific primers targeted to the spike gene, we have discovered nine samples which were positive in the PCR screening, with a continuous positivity for three years, which are 2/48 in 2015, 1/61 in 2016, and 6/157 in 2017.

We recovered two genome sequences from two strains of Grismo virus. The genomes of strains Grimso215 and Grimso2306 shared 95.6% sequence identity at the nucleotide level, with 1338 site differences. This divergence is notably higher than the expected differences based on a typical substitution rate for coronaviruses of 0.001 substitutions per site per year [16,17], which under Poisson distribution predicts an accumulation of 61–121 substitutions over three years. This observation suggests that either multiple strains of Grimso-like viruses are co-circulating in bank voles in Grimsö or that these viruses are transmitted regularly to bank voles from other species. One could also contemplate that the observed divergence could be associated with the temporal fluctuations in bank vole population density, leaving room for an increased viral transmission in a cyclic peak preceded by population turnover, similarly as previously described for Puumla Hantavirus dynamics in bank voles [18]. Nevertheless, with a prevalence of around 3.4% (9/266), we hypothesize that Swedish bank voles are competent hosts for the Grimso virus.

The wild animals provide pools of divergent virus species for interspecies transmission. The studies on the discovery of animal CoVs have been especially important since the emergence of human coronaviruses. The hidden diversity of CoVs has been explored in at least 37 rodent species distributed in Asia and Europe [3,4,15,19,20,21]. These studies point out that rodent CoVs have undergone frequent recombination and cross-species transmission events [15,20]. Previous studies found distinct CoVs in bank voles based on the partial sequences of the RdRp gene in the UK, Poland, Germany, and France [3,4]. Our phylogenetic analyses based on complete sequences of ORF1b, S, and N genes, as well as a partial RdRp gene, all suggested that bank voles carry one more divergent CoV, Grimso virus. Together, these observations suggest a relatively broad geographic distribution of CoVs in bank voles in Europe, which is indicative of possible long-term host–virus association. Furthermore, as coronaviruses closely related to the Grimso virus have been detected in bank voles elsewhere in Europe, it further supports that this divergent coronavirus infects and circulates in Swedish bank voles.

To understand the zoonotic risks of Grismo virus, we analyzed the protein domain of the spike protein and hemagglutinin esterase. Due to the high divergence and the lack of isolated live virus, we could not yet identify the host receptor usage for this novel rodent CoV. However, we cannot neglect the zoonotic potential of Grimso virus to livestock or humans. Together with mapping and continuous monitoring of the Grimso virus, further studies will aim to isolate the virus and assess the pathogenic profile.

## 5. Conclusions

We identified a highly divergent *Embecovirus,* named the Grimso virus, in Swedish bank voles. Our analyses suggest that multiple distinct viral strains co-circulate in this population, although further investigation will be necessary to fully understand the transmission ecology. Furthermore, we found that closely related coronaviruses are broadly distributed across Europe and exclusively associated with bank voles and other vole species, indicating that bank voles are likely natural reservoirs of the Grimso virus. While the potential threat posed by the virus to human and animal health is unknown, our findings underscore the importance of longitudinal surveillance of CoVs in wild rodents in advancing current knowledge on the ecology of CoVs in reservoir populations.

## Figures and Tables

**Table 1:** Identity (%) of Grimso215 strain on the genes across the genome (at nucleotide/amino acid levels) as compared to the representatives of known rodent CoVs.

	3CLpro	RdRp	ORF1ab (191–13,360) (13,684–21,402)	S (24,036–28,001)	E (28,674–28,925)	M (28,935–29,615)	N (29,625–31,019)
Grimso2306 (OM373091)	97.9/99.7	94.8/95.2	91.3/95.4	94.6/99.8	98.8/100	98.4/100	99.4/98.9
Rat coronavirus Parker (NC_012936)	71.4/74.9	78.1/86.3	65.3/51.8	54.6/45.3	61.7/53	66.5/66.5	57.8/52
HCoV_HKU1 (NC_006577)	71.2/73	79.5/84.4	66.2/51.2	55.3/45.2	59/46.3	68.4/65	55.7/49.7
Longquan708 (KF294372)	70.2/74.6	79.8/86.6	66.4/53.9	55/45.7	61.3/53	68.4/69.2	57.7/53.3
Longquan723 (MT820630)	73.4/76.6	78.3/85.8	65.6/52.4	54.6/45.5	65.7/56.6	67.1/68.3	57.6/53.7
HCoV-OC43 (KF530086)	72.2/74.9	79.6/85.3	67.1/53.8	56.1/45.2	66.1/59	68.6/66.5	58.7/54.6
RtMruf_JL2014 (NC_046954)	72.2/73.6	79.2/86.2	67.5/54.9	56.2/46.5	66.9/56.6	66.8/67.4	58.6/55.2

## Data Availability

Genbank sequence accession no.: OM373090 Grimso215, OM373091 Grimso2306.

## Supplementary Materials

The following supporting information can be downloaded at: https://www.mdpi.com/article/10.3390/v14061205/s1, Figure S1: Conserved domains on the hemagglutinin esterase (A) and spike (B) of Grimso virus.
