# Supplementary figures and images for: Discovery of a Novel Coronavirus in Swedish Bank Voles (Myodes glareolus)
Source: Viruses. 2022 Jun 1;14(6):1205. doi: 10.3390/v14061205 (PMC9230040; doi:10.3390/v14061205)

A

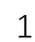

Supplement: Supplementary file 1 [file viruses-14-01205-s001.zip › viruses-1715217-Supplementary.pdf]
